# Differences in Perceptions of Health Information Between the Public and Health Care Professionals: Nonprobability Sampling Questionnaire Survey

**DOI:** 10.2196/14105

**Published:** 2019-07-03

**Authors:** Anat Gesser-Edelsburg, Nour Abed Elhadi Shahbari, Ricky Cohen, Adva Mir Halavi, Rana Hijazi, Galit Paz-Yaakobovitch, Yael Birman

**Affiliations:** 1 Health and Risk Communication Research Center and School of Public Health Haifa Israel

**Keywords:** health information-seeking, reading and understanding, quality criteria for health journalists, Web-based and newspaper health information sources, journalistic articles, public healthcare workers and the general public

## Abstract

**Background:**

In the new media age, the public searches for information both online and offline. Many studies have examined how the public reads and understands this information but very few investigate how people assess the quality of journalistic articles as opposed to information generated by health professionals.

**Objective:**

The aim of this study was to examine how public health care workers (HCWs) and the general public seek, read, and understand health information and to investigate the criteria by which they assess the quality of journalistic articles.

**Methods:**

A Web-based nonprobability sampling questionnaire survey was distributed to Israeli HCWs and members of the public via 3 social media outlets: Facebook, WhatsApp, and Instagram. A total of 979 respondents participated in the online survey via the Qualtrics XM platform.

**Results:**

The findings indicate that HCWs find academic articles more reliable than do members of the general public (44.4% and 28.4%, respectively, *P*<.001). Within each group, we found disparities between the places where people search for information and the sources they consider reliable. HCWs consider academic articles to be the most reliable, yet these are not their main information sources. In addition, HCWs often use social networks to search for information (18.2%, *P*<.001), despite considering them very unreliable (only 2.2% found them reliable, *P*<.001). The same paradoxes were found among the general public, where 37.5% (*P*<.001) seek information via social networks yet only 8.4% (*P*<.001) find them reliable. Out of 6 quality criteria, 4 were important both to HCWs and to the general public.

**Conclusions:**

In the new media age where information is accessible to all, the quality of articles about health is of critical importance. It is important that the criteria examined in this research become the norm in health writing for all stakeholders who write about health, whether they are professional journalists or citizen journalists writing in the new media.

## Introduction

### Searching for Web-Based Health Information

In the last decade, the internet has become a powerful instrument for searching for health information because it provides the opportunity to access information from varied and diverse sources [1]. According to current estimates, over 4 billion people have access to the internet [2]. An analysis of global internet use indicates that Israelis are second among the top ten countries worldwide in time spent online among individuals age fifteen or above [3]. Israelis use the internet more than Americans and Europeans [4] and spend the most time on the internet among global users [5].

As for science and health-related information, Israelis exhibit high levels of interest in science, with 62% of the public stating that knowing about science and technology in their everyday life is a necessity [6]. Moreover, polls have documented that health-related news is the most popular news topic among Israelis [7]. A recent study of otolaryngology patients found that Israelis turn to the internet as a source of health information significantly more than to books or newspapers [8].

Numerous studies have focused on factors that influence users in searching for health information. One such factor is health literacy [9-13]. Low health literacy is related to the limited ability to search, understand, and assess Web-based health information [14-16]. In contrast, high health literacy leads to more frequent Web-based searches for medical information [9,12].

Other variables found to influence the preference for internet sources include socioeconomic variables [13,17-19], cultural aspects [20,21], age variables [22-24], gender [25-28], and individual’s level of self-efficacy [27,29,30].

One of the main consequences of searching for health information is its impact on actual behavior [31,32]. Some studies have indicated a connection between information seeking and actual behavioral improvement [33,34], with effective searching for Web-based health information leading to positive outcomes such as an improved understanding of medical conditions, an improved understanding of treatment options, informed decision making, and effective stress reduction [35-39]. On the contrary, Web-based health information can also contain misinformation and disinformation and can influence the behavior of people from various health fields [16,40-42].

### Traditional Information Sources Versus Web-Based Sources

Before the digital age, the general public depended heavily on health organizations and official sources for health information. The digital revolution provided new alternatives that enabled laypersons to rely on additional and alternative information sources and to self-manage their information [43-45]. The internet revolution has made it possible for people to take an active part in their medical care and manage their daily health needs [46]. This process has led to a shift in the perceived role of the public, from passive recipient to active consumer of health information [16,45,47-49].

Web-based media constitute an important source of health-related information [50,51], as well as a platform for discussing and sharing personal experiences, opinions, and concerns regarding illnesses and treatments [49,52-57]. Furthermore, the internet serves as a democratic, accessible, and interactive source of diverse information, thus enabling patients to make informed decisions [58,59]. Seeking and sharing Web-based information provides people with social support while enabling them to maintain their anonymity [60,61].

As a result of this revolution, health information no longer belongs exclusively to health professionals but rather is also accessible to the general public [22,48,62]. Thus, patients tend to bring information from Google to their appointments to discuss with their doctors so that the patient–physician discourse has changed from one-directional to two-directional communication [10,63-65]. During consultations with their doctors, patients seek to verify the information they obtained from other sources. After a doctor’s appointment, some patients continue to search for information as a second opinion to verify the information they received from their doctor [18,62,66,67]. The reasons to continue searching information are as follows: the use of medical terminology in the physician–patient discourse impedes the patient’s understanding [50] and the short duration of physician–patient encounters leads patients to seek other information sources to find answers to questions that remain open. These and other factors often lead the public to doubt the credibility of physicians and to consume medical information from Web-based sources [16,27,50].

In contrast, other studies show that despite the internet revolution, doctors are still considered the main source of reliable information [22,68-71]. Some studies indicate that searching for Web-based information can improve physician–patient communication [72]. Moreover, some patients do not see the internet as replacing the doctor but rather as another resource that can help them better understand medical recommendations [73]. Some studies indicate that consuming Web-based information also increases the public’s reliance on medical professionals [74] for 2 main reasons: (1) the low reliability of the information on social networks [14,18,63,75-79] and (2) the low level of health literacy among the public, which makes it difficult for patients to understand and integrate health information and motivates them to turn to physicians as reliable sources [80-82].

In addition to doctors, before the digital revolution, the main agents through which the public obtained medical information were traditional media sources (eg, television and the press) [22,83-87]. Journalists who write about health acted as middlemen in communicating information to the public [16,42,88,89]. In today’s age of new media, professional journalists constitute a new voice in the discourse, alongside citizens who also define themselves as journalists [16,42,90]. Nevertheless, health journalists still play an important role because they are considered to be professionals by the general public, which continues to read their Web-based articles [16,88,91]. For example, the study by Pew Research Center reported that 95% of all new information being disseminated via news media came from old media—especially newspapers [87]. According to Walsh-Childers et al (2018), because patients become increasingly involved in the direction of their health care:

...health journalism will likewise increase in salience for audiences as an education source. The current climate encourages a level of patient involvement in medical decision making that requires health care consumers to have a much better understanding of the benefits, harms, and costs of all options available to them.

16

The literature analyzing journalistic quality points to the problematic nature of transmitting reliable information. Numerous studies over the past decade have found many problems in the media coverage of medications and medical treatments. These problems primarily emerge in the tendency toward sensationalism and over-enthusiasm in describing medications and medical technologies by placing excessive emphasis on their benefits while ignoring or hardly mentioning their risks, side effects, or costs [92-98]. The literature also found that journalists rely increasingly on websites and press releases from the medical industry and health organizations, which can result in the public perceiving professional journalism as biased and lacking credibility [42,99-101].

In light of the aforementioned changes, it is important to examine how the public in general and health care workers (HCWs) in particular assess the quality of the journalistic articles they read.

### The Quality of Health Information

Retrieving Web-based information often leads to misinformation or disinformation [102]. The quality of the information offered on the internet varies, ranging from evidence-based scientific data quoting scientific research and clinical experiments to questionable information that could imperil the individual’s health. Therefore, the challenge in searching for Web-based information and on social networks lies in the difficulty in finding sound, valid, and reliable information [103-105].

The proliferation of Web-based health information has led to a rise in the number of studies analyzing the quality of the published information. The first such study, published in 1997 by Impicciatore et al, evaluated the accuracy and integrity of the information on a website on fever management in children. According to the authors, out of 41 websites, only 4 provided full and accurate information about the subject [106]. This historic study provided a framework for subsequent studies of information quality. Accordingly, measurement tools were developed that usually included the following parameters: accuracy, completeness, readability, accountability, and technical criteria [103]. Additional specific parameters were added according to the subject of interest, for example, Eysenbach’s 6 criteria [103], the WebMedQual Scale [107], and Godin’s Quality Assurance Rating Tool for Internet Health Sites [108].

In recent years, these measurement tools and others have been used to assess information quality in different areas of medicine and health. All the studies point to the need to create reliable websites, improve information quality, improve access, increase oversight of Web-based medical information, and manufacture and distribute sound and customized materials [109-113]. Furthermore, websites have emerged that are dedicated to improving information quality for health writers. For example, the Health News Review website is an Australian-based website that aims to improve the public dialogue about health care by helping readers critically analyze health care news.

In general, most of the relevant literature in the new media age has focused on the reasons for seeking medical information, on diverse criteria that experts consider important to have on websites, and on the consequences of information seeking, whereas few studies have examined how the public reads and understands health information or what the public considers to be reliable.

This study sought to examine not only the ways in which the general public seeks information but also how the public understands this information and what information sources it sees as reliable. In addition, in view of the ongoing role of the press even in the new media era, the study also examines whether HCWs and the general public are capable of identifying quality criteria that influence behavioral intentions.

### Objectives

This research had 2 main objectives:

1. To examine how HCWs and the general public seek out, read, and understand health information.
2. To examine perceptions among HCWs and the general public regarding criteria for judging the quality of journalistic articles.

The specific objectives included the following: (1) to examine the differences between the general public and HCWs in how they seek and read information and how this new information influences their behavior and (2) to examine the criteria readers use to determine the quality of an article written by a journalist.

Comparing HCWs with the general public is based on the following rationale: As part of their daily work routine, HCWs are required to read and understand up-to-date information to be able to answer questions posed by the public. In addition, the public expects HCWs to answer questions about the information it encounters [10,16,63-65,72,114,115]. This raises questions regarding whether HCWs are able to discern the quality of journalistic information and whether they can refer the public to tools or criteria that can help in assessing the quality of articles appearing in the press.

The study is based on the following hypotheses:

1. The general public and HCWs will indicate that they will change their behavioral intentions after being exposed to health information.
2. The general public and HCWs will seek more health information from social networks than from scientific articles published in international journals.
3. The general public and HCWs will perceive scientific articles published in international journals as more reliable than information from social networks.
4. The general public and HCWs will be partially aware of the components that determine the quality of journalistic articles.
5. HCWs will perceive the criteria for determining the quality of health articles as more significant than will the general public.

## Methods

A survey was distributed to Israeli HCWs and members of the public via 3 social media outlets: Facebook, WhatsApp, and Google+. A total of 979 respondents participated in the online survey via the Qualtrics XM platform. The research was approved by the Faculty of Social Welfare and Health Sciences Ethics Committee for research with human subjects at the University of Haifa (Approval no. 266/18).

### Study Design

#### Sampling

Our sample was designed using Qualtrics XM online survey software as it provided rapid and efficient distribution of an interactive online questionnaire (see Multimedia Appendix 1) to our research population (HCWs and the general public in Israel). We used the self-selection in Web survey method of nonprobability sampling [116] to recruit participants through posts on social networks asking the general public (over the age of 18 years) to answer the survey.

Development of the Questionnaire and Research Procedure

##### Stage 1: Building the Questionnaire

The questions were based on a literature review in the field of health information seeking and on HealthNewsReview.org, an Australian website (https://www.healthnewsreview.org/) designed to rank health articles according to quality criteria. The questionnaire consisted of 3 parts. The first part asked the participant for sociodemographic details.

The second part included questions about information searching and reliability attributed to health information sources (eg, “Where do you usually search for information?” and “Which of the aforementioned information sources (social networks, health organization websites, human sources, web-based newspapers, public healthcare workers) do you consider the most reliable?”). Respondents were also asked about health issues that concern them: “What main health area usually interests and concerns you; what type of health information do you search for and read about?” Respondents chose from a list of medical topics: nutrition, physical activity, illnesses, medications, vaccinations, alternative therapies, safety, environmental exposure, and other. Questions also focused on how the public reads health articles (eg, “Do you read the whole article or just parts of it?”) and the impact of the information on behavior change (eg, “If you encounter health information which seems important to you, to what extent would you change your behavior after learning about this?”). An example is information published by the World Health Organization indicating that processed meat raises the risk of cancer.

The third part of the questionnaire focused on what determines whether a health article is of high quality. Respondents were asked to rank the list of criteria they were given (see Table 1) on a 5-point Likert scale ranging from 1 (completely agree) to 5 (completely disagree).

**Table 1:** Importance of health information criteria to health care workers and to the general public.

Criteria	Respondents^a^	
	Health workers	General public
The article also presents the drawbacks of the intervention.	4.27	4.13
The "tone" of the article is more scientific than marketing.	4.18	3.96
The article presents alternatives to medical intervention.	4.17	4.05
The article is based on a number of articles.	4.16	3.86
Details of the study.	4.09	3.87
The article cites results from an article from an academic journal.	4.06	3.78
Presentation of quantitative findings and not personal stories.	4.04	3.84
The article presents a scientific controversy in the field.	4.01	3.80
The article notes the availability and accessibility of treatment to the general public.	3.97	3.94
The article explains and simplifies professional concepts.	3.96	4.03
The article presents an opposing professional opinion.	3.93	3.73
The article presents existing conflicts of interest of the researchers.	3.76	3.51
The article presents information that has implications for policy.	3.75	3.45
The article presents a response by the regulator.	3.72	3.56
The article presents the findings even in the event that science indicates that there are no unequivocal answers.	3.68	3.32

^a^Respondents were asked to rank the list of criteria they were given on a 5-point Likert scale ranging from 1 (completely agree) to 5 (completely disagree).

##### Stage 2: Criteria Validation Process

Before distributing the questionnaire to each population group, we analyzed the validity of the criteria using a focus group consisting of 25 students and 7 researchers at the School of Public Health (University of Haifa, Israel), who rated 3 health articles according to the criteria. We measured their overall percentage of agreement, as well as Krippendorff alpha (representing the level of agreement between coders beyond mere chance) for each answer [117]. Overall, for all the criteria, the between-coder agreement was satisfactory (alpha=.79; 92%).

##### Stage 3: Pilot Survey for Content Validation

In the pilot, we distributed the questionnaire to 80 Arab and Jewish members of the general public and HCWs. The participants were asked to provide feedback on the questionnaire’s content. Subsequently, we focused on adjusting the questions to make them more culturally sensitive. For example, we used the word regulator in Hebrew, but as that term is not used in Arabic, we modified the word to policy. Similarly, conflict of interest is not a familiar concept in Arabic, so in the interest of clarity, an example was provided. Furthermore, as the general public did not always understand the full meaning of a question, we added clarifying examples. For instance, for the item stating that “The article gives possible solutions for different medical issues”, we gave examples of a possible solution (eg, making a lifestyle change instead of taking medicine to lower high blood pressure).

##### Stage 4: Running the Study

To recruit as many participants as possible, we used intensive sampling in the first step and distributed the questionnaires via social media platforms (WhatsApp, Facebook, and Instagram). After this initial sampling, we continued to recruit participants through snowball sampling [118] to reach enough participants among HCWs by distributing the questionnaire via specialized HCW Web-based forums and by directly asking them to distribute the questionnaire to additional HCWs they knew.

At interim meetings during the survey, we monitored the social demographic variables and noted a lack of young men among the general public and the HCWs who responded to the survey. As our audience was a deliberate sample, we looked for ways to distribute the survey to more HCWs and turned to health forums. By means of diffusion, the survey was distributed from our inner circles to extended circles.

### Analysis

To check whether people intended to change their behavior after being exposed to health information, we used a chi-square test in which the answers are reduced to 3 levels (not at all or to a small extent, to a medium extent, and to a high to very high extent). Chi-square tests for independence were conducted to examine the differences between HCWs and others with regard to information seeking, sources of information, source reliability, and the manner in which the information was read. Wilcoxon Rank-Sum tests were used to examine the differences between HCWs and the public regarding behavioral change following exposure to information and the criteria for a high-quality article.

Regarding the quality criteria for articles, separate chi-square tests were conducted for each criterion to examine the differences between HCWs and others. To avoid the inflation of a type I error owing to multiple testing, adjusted P values were calculated using the false discovery rate method.

## Results

A total of 979 respondents participated in the survey. The vast majority of the respondents (96%) were below retirement age (<66 years) and female (76%), almost half were Jewish (49%) and somewhat fewer (42%) were Muslim. Table 2 depicts the respondents’ sociodemographic and health status information.

**Table 2:** Sociodemographic and health status characteristics (n=979).

Sociodemographic characteristics and category		n (%)
**Gender**		
	Male	232 (23.7)
	Female	747 (76.3)
**Age (years)**		
	<29	363 (37.1)
	30-45	397 (40.6)
	46-65	177 (18.1)
	66+	42 (4.3)
**Ethnicity**		
	Jewish	481 (49.1)
	Muslim	410 (41.9)
	Christian	65 (6.6)
	Druze	12 (1.2)
	Other	11 (1.1)
**HCWs^a^**		
	Yes	363 (37.1)
	No	616 (62.9)
**Suffering from a chronic disease**		
	Yes	203 (20.7)
	No	776 (9.3)
**Child suffers from a chronic disease**		
	Yes	145 (14.8)
	No	834 (85.2)

^a^HCWs: health care workers.

### Behavioral Intentions Following Exposure to Health Information: General Public Versus Health Care Workers

Respondents were asked about the extent to which they would change their behavior after receiving health information of personal importance: “If you encounter health information that seems important to you, to what extent would you change your behavior following your exposure to this information?” The research findings indicate that more than half the HCWs and more than half the respondents from the general public reported they would change their behavior to a large or very large extent (Table 3).

**Table 3:** Intention to change behavior after receiving health information of personal importance. Question: If you encounter health information that seems important to you, to what extent would you change your behavior following exposure to this information?

Respondents	Intent to change			
	Not at all or to a low extent, n (%)	To a moderate extent, n (%)	To a large or very large extent, n (%)	Total
HCWs^a^	20 (5.51)	132 (36.36)	211 (58.13)	363
GP^b^	66 (10.71)	220 (35.71)	330 (53.57)	616
Total	86 (8.78)	352 (35.96)	541 (55.26)	979

^a^HCWs: health care workers.

^b^GP: general public.

### Seeking Health Information and Perceived Reliable Sources—the General Public Versus Health Care Workers

Table 4, Table 5 and Table 6 show where the general public and HCWs search for health information. In comparison with the general public, HCWs mainly search on health organization sites and in academic articles, as they consider academic articles more reliable. The general public seeks more information from social networks and Web-based newspapers and considers social networks, human resources, and HCWs to be more reliable.

**Table 4:** Seeking information and source reliability: comparison between health care workers and the general public.

Sources for health information		Respondents, %	GP^a^, %	HCWs^b^, %	Chi-square (*df*)	*P* value	Adjusted *P* value^c^
**Social networks**						
	Where do you usually search for health information?	30.34	37.50	18.18	40.33 (1)	<.001^d^	<.001^d^
	Which source is most reliable in your opinion?	6.13	8.44	2.20	15.45 (1)	<.001^d^	<.001^d^
**Health organizations**						
	Where do you usually search for health information?	33.40	27.11	44.08	29.56 (1)	<.001^d^	<.001^d^
	Which source is most reliable in your opinion?	44.13	43.02	46.01	0.83 (1)	.36	.36
**Human sources**						
	Where do you usually search for health information?	4.80	5.84	3.03	3.96 (1)	.05	.06
	Which source is most reliable in your opinion?	5.82	7.79	2.48	11.76 (1)	<.001^d^	<.001^d^
**Academic articles**						
	Where do you usually search for health information?	17.06	12.82	24.24	21.05 (1)	<.001^d^	<.001^d^
	Which source is most reliable in your opinion?	34.32	28.41	44.35	25.76 (1)	<.001^d^	<.001^d^
**Public health care workers**						
	Where do you usually search for health information?	5.52	5.68	5.23	0.09 (1)	.77	.77
	Which source is most reliable in your opinion?	8.27	10.71	4.13	13.04 (1)	<.001^d^	<.001^d^
**Web-based newspapers**						
	Where do you usually search for health information?	8.89	11.04	5.23	9.51 (1)	.00^e^	.00^e^
	Which source is most reliable in your opinion?	1.33	1.62	0.83	1.11 (1)	.29	.35

^a^GP: general public.

^b^HCWs: health care workers.

^c^False discovery rate.

^d^*P*<.001.

^e^*P*<.05.

**Table 5:** Primary information source and perception of reliability (percentage of health care workers).

Information source	Source used to search for information	Most reliable source
Health organizations	44%	46%
Academic articles	24%	44%
Social networks	18%	2%
Public health workers	5%	4%
Human sources	3%	2%
Web-based newspapers	5%	1%

**Table 6:** Primary information source and perception of reliability (percentage of general public).

Information source	Source used to search for information	Most reliable source
Health organizations	27%	43%
Academic articles	13%	28%
Social networks	38%	8%
Public health workers	6%	11%
Human sources	6%	8%
Web-based newspapers	11%	2%

### Health Care Workers Perceive the Criteria for Quality Health Articles as More Significant Than the General Public

The differences between HCWs and the general public in their perceptions of the importance of health information quality criteria are statistically significant, with the exception of 2 criteria: the article explains and simplifies professional concepts and the article notes the availability and accessibility of treatment to the general public. Inclusion of the criteria in the articles is more important for HCWs than for the general public (Table 1).

The comparison between the criteria rankings of the HCWs and those of the general public shown in Table 1 indicates that both groups ranked the following criteria at the top of the list: intervention drawbacks; tone more scientific than commercial; offers alternatives to medical interventions; based on several articles; and details of the study. Among the HCWs, the importance of citing results from academic articles was next on the list, whereas the general public ranked this criterion in the tenth place, instead ranking presentation of quantitative findings and not personal stories in the sixth place. Both the HCWs and general public ranked conflict of interest at the bottom of the chart (not shown in Table 7).

**Table 7:** Ranking of top 6 health information criteria among health care workers versus the general public.

Group and criteria		5-point Likert scale ranging from 1 (completely agree) to 5 (completely disagree)	*P* value^a^
**HCWs^b^**	
	Drawbacks of the intervention	4.27	.01^b^
	Tone more scientific than commercial	4.18	.00^c^
	Alternatives to medical interventions	4.17	.04^c^
	Based on several articles	4.16	<.0001^d^
	Details of the study	4.09	.00^c^
	Cites results from academic articles	4.06	<.0001^d^
**General public**	
	Drawbacks of the intervention	4.13	.00^c^
	Alternatives to medical interventions	4.05	.04^c^
	Tone more scientific than commercial	3.96	.00^c^
	Details of the study	3.87	.00^c^
	Based on several articles	3.86	<.0001^d^
	Presents quantitative findings and not personal stories	3.84	.00^c^

^a^Wilcoxon Rank-sum Test

^b^HCWs: health care workers.

^c^*P*<.05.

^d^*P*<.001.

## Discussion

### Principal Findings

The new media age has changed the way people seek and consume health information [1,46]. The purpose of this study was to investigate not only how people search for Web-based and newspaper health information but also how they read and understand this information and what criteria they use to assess the quality of journalistic articles. It is important to examine how people read, understand, and assess the quality of journalistic health articles because health information can influence the way people shape their healthy lifestyles [33,34,119,120].

The findings of this study confirm the importance of this examination. When participants were asked whether they intended to change their behavior after being exposed to health information, more than 30% responded that they would make moderate changes to their behavior and 50% responded that they would make extensive changes.

The findings indicate that HCWs focus their search for health information on health organization sites and in academic articles, whereas the general public tends to search more on social networks and Web-based newspapers. This finding can be explained by the HCWs’ professional context [121]. Public HCWs are accustomed to interacting with the health system on a daily basis and naturally search more on health organization websites [122,123]. Similarly, it is reasonable to assume that in the course of their work, professionals are more likely to use academic articles than the general public [124].

Moreover, the difference in choice of information sources between HCWs and the general public can also be explained by the level of health literacy. It is reasonable to assume that HCWs have a higher level of health literacy and are more capable of processing and understanding complex medical information than the general public, leading them to place more trust in scientific sources than in social networks or information available on the internet [12,125].

Furthermore, when participants were asked what they consider to be a reliable source, HCWs found academic articles more reliable than did the general public, which found social networks [55,58,59], human sources, and HCWs to be more reliable [68,74].

As for information seeking, HCWs found academic articles based on scientific facts to be more reliable than information from social networks. Moreover, they considered information based on scientific evidence from academic journals to be more reliable and to have a more scientific than commercial tone. Thus, differences between HCWs and the general public can be explained based on the training that HCWs undergo.

Nevertheless, when we examined each group separately, differences emerged within each group. Even though HCWs indicated that academic articles are the most reliable, they tended to search more for information on social networks despite considering them very unreliable [126]. We found a similar discrepancy among the general public, which considered health organizations and academic articles to be very reliable or reliable sources yet used them infrequently to search for health information, preferring social networks, which they considered unreliable. The research findings confirm our hypothesis that both HCWs and the general public search for health information on social networks more than they do in sources they consider reliable.

This discrepancy between perceptions and actual behavior is in line with studies indicating that there are situational factors whose influence is stronger than mere attitudes. According to Wicker (1969) [127], even though participants believe that health organizations and academic articles are more reliable sources than social networks, in practice, most of them search in sources they consider to be less reliable.

The following 3 explanations attempt to answer why the public seeks information from social networks more than from other sources. First, health organizations do not provide responses to the public’s questions. That is, they conduct a monologue rather than a dialogue, leading the public to seek more information on social networks. The world of social media has generated a radical transformation in the relationship between government organizations and the public. Social media have changed the monologue to a dialogue in which anyone with information and access to communications technology can be a content creator and communicator [128]. Over the past decade, leading international health authorities, health ministries, and local governments have invested financial and human resources to narrow the gaps between the authorities and the public, thus increasing the authorities’ presence on social media. Despite this impressive transformation, the use of social media by organizations is still in its infancy. Although the literature indicates that health authorities use social media, it also shows that this use is still very limited, as these tools serve primarily for mass information dissemination (similar to traditional mass media) instead of for 2-way communication [129-132].

Second, as health organizations do not exercise complete transparency in conveying information, the public turns to social networks to fill in the missing information. It is important to note that alongside disinformation deliberately conveyed by stakeholders, most of the discourse on social media stems from people’s desire to obtain additional information, which is sometimes not fully conveyed by the health organizations [43].

Third, among the general public as well as among HCWs, decision making on health matters entails a combination between the automatic emotional system and the rational system. Thus, it is no wonder that despite being aware that social networks are likely to contain misinformation or disinformation, people continue to seek information there [133]. Studies of the health behavior of public HCWs found that they shared the same concerns and barriers as the general public [43,134-137]. Neither public HCWs nor the general public rely only on analytical or evidence-based information (academic articles) when searching for health information, but also seek information based on experience and emotions, both of which are found mostly on the social networks.

In summary, we proposed several possible explanations for the discrepancy we found between what the public and HCWs believe to be reliable and where they actually search for information in practice.

As for the research findings about what criteria the general public uses to judge the quality of journalistic articles compared with the criteria used by public HCWs, statistically significant differences were found between the importance of the criteria (except for 2), indicating that HCWs attributed more importance to the criteria than the public.

In addition, when we examined the 6 criteria that were most important to the general public and to HCWs, we found that 4 were important for both groups: drawbacks of the intervention, a tone that is more scientific than commercial, alternatives to medical interventions, and details of the study. These criteria indicate that the public values providing full information about the negative impacts or limitations of medical interventions as well as existing alternatives, information often absent from the media coverage. Studies have found that media coverage of medications and medical treatments is problematic, primarily in its tendency to provide sensational and an overly enthusiastic coverage of drugs and medical technologies and to emphasize the benefits excessively while ignoring or hardly mentioning the risks, side effects, and costs [93,94,96,97,98] or the limitations of scientific studies advocating the efficacy of these drugs.

In addition to the 4 aforesaid criteria cited both by public HCWs and by the general public as indications of information quality, 2 specific criteria emerged as important to the public. One of them is that the article should mention treatment availability and accessibility. This finding can be explained by the public's wish to know whether the treatment or medication mentioned in the article is accessible to it. For marketing reasons, press reports often mention medications and interventions that are not accessible to the public [92]. A second criterion valued by the public is that the article should simplify professional concepts. The importance of communicating professional information in understandable and clear language is a basic principle cited in the health communication and risk communication literature [138,139]. The mental models approach [140] also emphasizes the importance of understanding and simplifying professional concepts for the general public. Conversely, a criterion the public did not consider important was citing academic sources.

The findings also indicate that both HCWs and the general public ranked conflict of interest at the bottom of the list. Studies indicate that for years journalists have relied on information provided to them by organizations and the pharmaceutical industry rather than looking for quotes from academic sources themselves [99,100]. Several scholars have warned that journalists often fail to disclose the funding sources supporting the research, the investigators’ financial conflicts of interests, and all the sources interviewed [95,141-143]. Owing to such potential conflicts of interest, reporting a study’s limitations, funding sources, and financial ties is of great importance [144]. The Statement of Principles of the US Association of Health Care Journalists calls on journalists to disclose relevant conflicts of interest in their sources as a routine part of their work [145]. Yet, it seems that more often than not, journalists do not report such conflicts of interest [97,98,145].

The public may have become used to reading information in such a way that it does not look for citations from academic sources but relies on the author’s integration or summary. In addition, the lack of discussion about the importance of exposing conflicts of interest leads both public HCWs and the general public not to attach adequate importance to this issue. The importance of including scientific articles and assessing their quality and the importance of disclosing conflicts of interest are criteria whose absence can produce misinformation, partial information, or disinformation that affect the public’s decision making.

### Limitations

This study is not a representative sample of the general population of Israel. It used nonprobability sampling and measuring and was therefore vulnerable to selection bias from the outset.

Furthermore, in this study, we did not check the impact of several variables that might affect health-information searching behavior, both of the general public and of health workers, such as age, gender, personal relevance, level of health literacy, and the individual's reasons for the health information search. Also, as in any study checking behavioral intentions and actual behavior, this study is vulnerable to information bias as the result of biased reporting by the respondents. Our overall goal was to reach the specific target audiences of the general public versus HCWs and compare them, even though it was not a representative sample of those 2 populations.

We took a number of steps to minimize sampling bias: (1) we used 3 different media channels, thus increasing the chances for randomization in this sample [116]; (2) we monitored the data once a week to insure sufficient professional and ethnic representation among the participants. For example, when we discovered that there was an insufficient number of HCWs, we posted on more medical forums. When we noticed there were not enough participants from the Arab sector, we appealed specifically to this population group and thus broadened the sample; and (3) we used snowball sampling according to which each participant gave the questionnaire to someone else from their group, enabling us to reach more people from the required population groups. As our study is based on a small subpopulation of HCWs, the choice of the snowball sampling technique seemed to be more appropriate than convenience sampling. In addition, the descriptive statistics suggest that we were able to achieve a diverse sample based on sociodemographic variables.

### Conclusions

The study findings point to disparities both among HCWs and among the general public in their information-seeking behavior and their evaluations of the reliability of the sources searched. To reduce these gaps, health organizations must provide attractive materials, make academic articles accessible, and improve their dialogue with the public. In addition, in the technological age, where information is accessible to all, the quality of articles about health is critically important. Making the criteria cited in this research the norm in health writing is important for all stakeholders who write about health, whether they are professional journalists or citizen journalists in the new media.

## Appendix

Multimedia Appendix 1 Questionnaire.

## Abbreviations

HCW: health care worker
