# Supplementary material for: Differences in Perceptions of Health Information Between the Public and Health Care Professionals: Nonprobability Sampling Questionnaire Survey
Source: J Med Internet Res. 2019 Jul 3;21(7):e14105. doi: 10.2196/14105 (PMC6639070; doi:10.2196/14105)
Supplement: Multimedia Appendix 1 [file jmir_v21i6e14105_app1.docx]

**Questionnaire: Reading information related to health issues**

**Do you have a prolonged illness or a health problem that has persisted or is expected to persist 6 months or longer?**

Yes/No

**Is there anyone from your immediate family (children/spouse/parents) who has a chronic disease?**

Yes/No

**To what extent does the health field interest you? (mark the correct answer)**

To a very large extent

To a large extent

To a moderate extent

To a little extent

Not at all

**Are you a member of a blog/forum related to a health site?**

Yes/No

**If yes, which one?**

**If you work in public health, how often do you search for health information regardless of your occupation? (If you do not work in public health you can skip to the next question)**

Once a day

Once a week

Once a month

Rarely

**If you encounter health information that you think is important, to what extent will you change your behavior as a result of exposure to this information? (For example, the World Health Organization published information in the media that processed meat increases cancer morbidity)**

To a very large extent

To a large extent

To a moderate extent

To a little extent

Not at all

**For the most part, what is the main area of health that interests and concerns you and that you will search for and read information about? (Please mark one answer)**

Nutrition

Physical activity

Diseases

Medicines

Vaccines

Alternative treatments

Safety

Environmental exposure concerning health

Other

**If you choose "other" please specify:**

**Where do you usually search for information on the subject you answered in the previous question?**

Social networks: Facebook/ Twitter/ Instagram/ Forums/ Blogs

Health organization sites: HMO/ Ministry of Health/ WHO/ designated health sites

Human sources: family/ friends/ co-workers

Academic articles

Public health workers (doctor, nurse, pharmacist, dietitian, etc.)

Online newspapers such as: YNET, Walla, PANET, Al-Arab

**Which of these sources is most reliable in your opinion? (please mark one answer)**

Social network sites

Health organizations

Human sources

Online newspapers

Public health workers

Academic articles

**Select the statement that best describes how you read a health article**

It's usually enough to read the title only

It's usually enough to read the title and the subheading (abstract)

I read the whole article

It's usually enough to read the title and the last paragraph

**For each of the following components, indicate the extent you believe this component determines that the article on health is high quality?**

Highly agree

Agree

Moderately agree

Agree to a lesser extent

Do not agree

- **The article is published in a newspaper that presents scientific research**
- **Details of the study are given (number of participants, clinical trial stages, influencing factors, etc.)**
- **The article provides quantitative findings (numerical / statistical) and not personal stories.**
- **The article also presents the drawbacks of the intervention (e.g., drug side effects)**
- **The article presents alternatives to medical intervention (e.g., lifestyle changes to treat hypertension rather than medication)**
- **The article cites results from an article from an academic journal**
- **The "tone" of the article is more scientific than commercial**
- **The article presents an opposing professional opinion**
- **The article notes the availability and accessibility of treatment to the general public**
- **The article presents existing conflicts of interest of the researchers (for example, who finances the research?)**
- **The article is based on a number of articles (systematic literature review)**
- **The article presents a scientific controversy in the field (if there is a scientific disagreement in the professional literature on the subject in question, the writer notes this)**
- **The article presents information that has implications for policy (for example, the information in the article affects decision-makers)**
- **The article presents the findings even in the event that science indicates there are no unequivocal answers**
- **The article presents a response by the regulator (relevant government ministries) and recommendations on how to act**
- **The article explains and simplifies professional concepts (from the research field and medicine)**
